# PGK1 represses autophagy-mediated cell death to promote the proliferation of liver cancer cells by phosphorylating PRAS40

**DOI:** 10.1038/s41419-022-04499-0

**Published:** 2022-01-20

**Authors:** Tianhua Zhang, Yuzhen Wang, Hongjiu Yu, Ting Zhang, Lianying Guo, Jie Xu, Xiaoqing Wei, Ning Wang, Yingjie Wu, Xiuli Wang, Lin Huang

**Affiliations:** 1 Department of Pathophysiology, College of Basic Medical Sciences, Dalian Medical University, 9 South Lvshun Road, 116044 Dalian, Liaoning P.R. China; 2 International Medical Center, The First Affiliated Hospital, Dalian Medical University, 9 South Lvshun Road, 116044 Dalian, Liaoning P.R. China; 3 Institute of Cancer Stem Cell, Dalian Medical University, 9 South Lvshun Road, 116044 Dalian, Liaoning P.R. China; 4 Molecular Medicine Laboratory, College of Basic Medical Sciences, Dalian Medical University, 9 South Lvshun Road, 116044 Dalian, Liaoning P.R. China; 5 Institute for Genome Engineered Animal Models of Human Diseases, Dalian Medical University, 9 South Lvshun Road, 116044 Dalian, Liaoning P.R. China; 6 Liaoning Provence Key Lab of Genome Engineered Animal Models, Dalian Medical University, 9 South Lvshun Road, 116044 Dalian, Liaoning P.R. China; 7 Department of Molecular Pathobiology, New York University College of Dentistry, New York, NY 10010 USA; 8 Department of Histology & Embryology, College of Basic Medical Science, Dalian Medical University, 9 South Lvshun Road, 116044 Dalian, Liaoning P.R. China

**Keywords:** Macroautophagy, Macroautophagy

## Abstract

Autophagy predominantly promotes cell survival by recycling cell components, while it kills cells in specific contexts. Cell death related to autophagy plays important roles in multiple physiological and pathological situations including tumorigenesis, and the mechanism needs to be defined further. PRAS40 was found to be crucial in various cancers, and phosphorylation was reported to be involved in autophagy inhibition in monocytes. However, the detailed role of PRAS40 in autophagy and the relationship to tumorigenesis remain largely unknown. Herein we screened the binding partners of PRAS40, and found that PRAS40 interacted with Phosphoglycerate kinase 1 (PGK1). PGK1 phosphorylated PRAS40 at Threonine 246, which could be inhibited by blocking the interaction. Both in vitro and in vivo results revealed that PRAS40 mediated PGK1-induced cell growth. By tracing the mechanism, we found that PGK1 suppressed autophagy-mediated cell death, in which PRAS40 was crucial. Thus PGK1 phosphorylates PRAS40 to repress autophagy-mediated cell death under normoxia, promoting cellular proliferation. The binding of PGK1 to PRAS40 was transferred to Beclin1 under hypoxia, resulting in the increase of Beclin1 phosphorylation. These results suggest a novel model of tumorigenesis, in which PGK1 switches between repressing autophagy-mediated cell death via PRAS40 and inducing autophagy through Beclin1 according to the environmental oxygen level. Our study is anticipated to be able to offer novel insights in understanding PGK1/PRAS40 signaling hyperactivated cancers.

## Introduction

The balance between cell survival and death is critical to homeostasis, in which autophagy plays an important role. Autophagy is an evolutionarily conserved mechanism for recycling intracellular parts to protect cells, while also killing cells under certain conditions [1]. People try to clarify the different mechanisms between both events. Although some believe that a little autophagy protects cells, whereas a lot induces death; a second signal delivered to an autophagy initiation machine may be necessary for cell death [2, 3]. Nevertheless, the regulation mechanism remains largely unknown. Autophagy-mediated cell death is a defined type of cell death related to autophagy, which is triggered by autophagy and involves a standard mechanism of cell death, such as apoptosis [1, 4].

Primarily determined as a binding protein of 14-3-3 [5], a substrate of AKT [6], and a component of mTOR complex 1 [6–10], PRAS40 (encoded by *AKT1S1*) has been frequently reported to play an important role in tumorigenesis. PRAS40 functions in cell survival, proliferation, apoptosis, senescence, metastasis, immunoregulation, and protein degradation in various species [6, 11–14]. Recently we demonstrated that PRAS40 is hyperexpressed in hepatocellular carcinoma (HCC), and its expression is negatively correlated to the survival rates of patients [15]. PRAS40 promotes cellular proliferation by deregulating apoptosis in several kinds of tumors [12, 13, 16], which might be mediated by p53 upregulation in a Ribosomal protein 11-mediated manner [17]. Phosphorylation is considered as the active form of PRAS40, lots of phosphorylation sites in the C-terminal region are responsible for growth factor stimulation and nutrients [18, 19], and Thr246 hyperphosphorylation has been found in the clinical specimens of HCC, prostate cancer, melanoma, and non-small cell lung cancer [13, 20, 21]. Although AKT activation is considered as the driver of Thr246 phosphorylation, other kinases including Rab11-family interacting protein 4 [22], Proviral integration site for Moloney murine leukemia virus-1 [23], phosphoinositide-mediated kinase 1 [24], and Dual-specificity tyrosine phosphorylation regulated kinase 3 [25] are also responsible for it. More details of the function and regulation of PRAS40 phosphorylation in cancer remain to be clarified. Uric acid priming led to the phosphorylation of AKT-PRAS40 as well as autophagy repression in monocytes [26], while the autophagic role of PRAS40 in cancer and the details of PRAS40 relating to autophagy need to be determined further.

PGK1 functions as either an ATP-producing glycolytic enzyme [27, 28] or a protein kinase [29–31], contributing to the oncogenic function. PGK1 overexpression has been detected in colon cancer [31], pancreatic cancer [32], HCC [27], and etc. Under glutamine deprivation or hypoxia, acetylated PGK1 phosphorylates Beclin1 to induce autophagy in tumorigenesis [29, 30]. However, more details related to the role of PGK1 in tumorigenesis and autophagy need to be elucidated, including under normal levels of oxygen or nutrient. In addition, the crosstalk of PGK1 and AKT/mTOR pathway has been reported before, whereas the mechanism through which PGK1 is involved remains elusive [28, 33].

We determined here the binding partners of PRAS40 by mass spectrometry following co-immunoprecipitation assay (Co-IP). PRAS40 interacted with and was phosphorylated by PGK1, promoting the suppression of autophagy-mediated cell death by PGK1 to contribute to tumorigenesis. This study would provide novel insights for understanding the role of PRAS40 and PGK1 in autophagy-mediated cell death, which could offer new clues to target cancers. Moreover, the phenomenon that PGK1 shifts the binding partner depending on oxygen level suggests a novel role of tumorigenesis, in which PGK1 switches between autophagy-mediated cell death and autophagy with different mechanisms, contributing to tumorigenesis in a different environment.

## Results

### PRAS40 interacts with PGK1

**Fig. 1:**
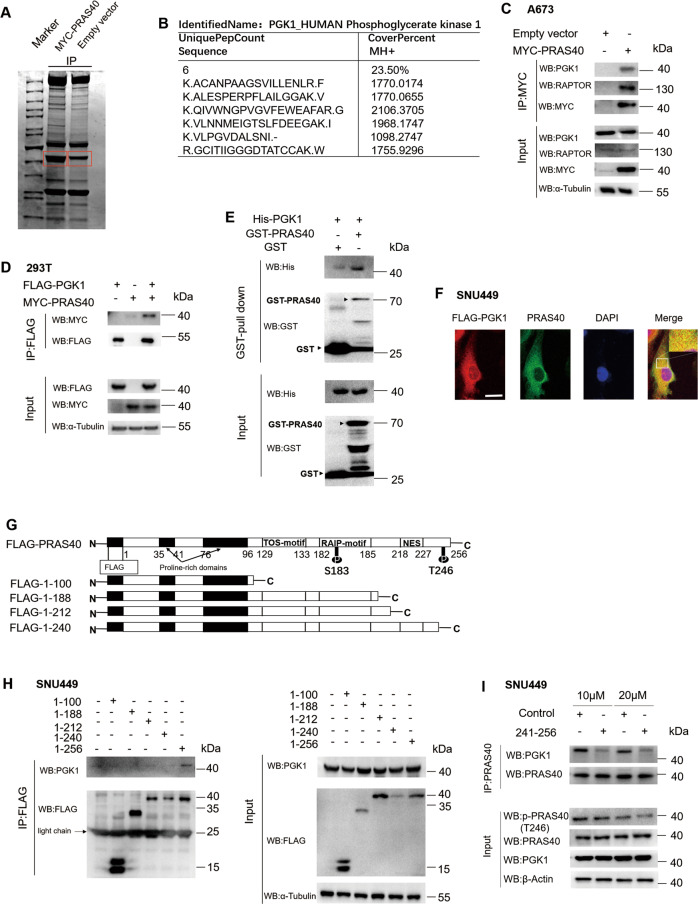
Interaction between PRAS40 and PGK1. **A–C** Co-IP with anti-Myc antibody. The bands with red boxes were cut out and applied to mass spectrometry (**A**). The unique peptides of PGK1 enriched in Myc-PRAS40-bound precipitates were clarified by mass spectrometric analyses (**B**). Western Blotting analyses using the indicated antibodies (**C**). **D** Co-IP followed by western blotting analyses with anti-FLAG antibody. **E** GST pull-down assay using GST or GST-PRAS40 together with His-PGK1. **F** Immunofluorescent staining with anti-FLAG and anti-PRAS40 antibodies in the cells introduced with FLAG-PGK1. Scale bar, 10 μm. **G** Schematic diagram showing the deletion mutants of PRAS40. **H, I** Co-IP followed by western blotting analyses with the indicated antibodies in the indicated cells. The cells were treated with peptide control or peptide 241–256 at the indicated concentrations **I**.

To investigate the function network of PRAS40, we screened the binding proteins of PRAS40 by Co-IP with anti-Myc antibody using cell lysates from empty vector- or Myc-PRAS40-transfected cells. On the SDS-PAGE loaded with the precipitates, the bands of both samples were compared. The most significant difference was observed in the bands framed with red boxes, which were cut out, trypsinized, and subjected to mass spectrometry (Fig. 1A). According to the results from mass spectrometry, 6 unique peptides of PGK1 (cover rate = 23.5%) were found to be enriched in Myc-PRAS40 positive cell lysate (Fig. 1B, S1). Western blotting revealed that PGK1 was present in the precipitates from Myc-PRAS40- but not empty vector-transfected cells as same as RAPTOR, a determined interacting factor of PRAS40 (Fig. 1C). Similar results were also obtained in HEK293T cells, HepG2 cells, and SNU449 cells (Fig. S2A-C). We next co-transfected FLAG-PGK1 and Myc-PRAS40 into HEK293T cells, the results showed that Myc-PRAS40 was present in the precipitates from FLAG-PGK1- but not empty vector-transfected cells (Fig. 1D). The results of GST pull-down assay showed that PGK1 was present in the precipitates of GST-PRAS40-bound sepharose but not GST-bound sepharose (Fig. 1E). In addition, the immunostaining results revealed that PRAS40 and PGK1 co-localized mostly in the cytoplasm (Fig. 1F, S2D). Thus PRAS40 binds PGK1.

### The region of amino acid 241–256 in PRAS40 mediates the interaction of PRAS40 and PGK1

To determine the region of PRAS40 necessary for binding PGK1, serial deletion mutants of PRAS40 were constructed (Fig. 1G). Western blotting following Co-IP revealed that PGK1 was present in the precipitates from the cells introduced with full-length PRAS40 (1–256). However, PGK1 was detected in none of the precipitates from the cells introduced with deletion mutants, as well as the cells introduced with empty vector (Fig. 1H).

To confirm that the binding domain in PRAS40 locates at the region of amino acid 241–256 further, we constructed a fusion peptide designed by linking a previously identified cell-penetrating peptide to peptide 241–256. After the addition of the chimeric peptide, we performed Co-IP with anti-PRAS40 antibody, and less PGK1 was detected in the precipitates, compared with that of control peptide-added cells (Fig. 1I, S2D). Therefore PGK1 interacts with PRAS40 through the region of amino acid 241–256.

### Kinase PGK1 phosphorylates PRAS40

To clarify the significance of the interaction of PGK1 and PRAS40, we used the peptide 241–256 to disrupt the interaction, and found that PRAS40 phosphorylation level was significantly downregulated (Fig. 1I, S2E, S3A).

**Fig. 2:**
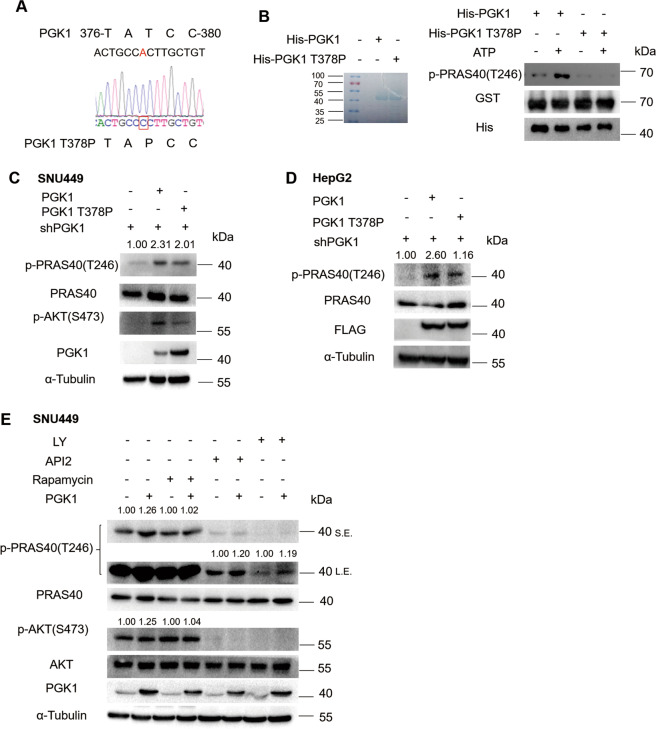
Correlation of PRAS40 phosphorylation with kinase PGK1. **A** Sequencing result of a kinase-deficient mutant PGK1 T378P. The mutated nucleic acid was framed with a red box. **B** Coomassie blue staining image for the gel loaded with purified His-PGK1 or His-PGK1 T378P (left panel). Kinase assays using GST-PRAS40 together with His-PGK1 or His-PGK1 T378P (right panel) followed by western blotting analyses with the indicated antibodies. **C–E** Western blotting analyses with the indicated antibodies. The cells depleted with PGK1 were introduced with empty vector, shRNA-resistant FLAG-PGK1, or FLAG-PGK1 T378P expression vector (**C, D**). The indicated cells were treated with rapamycine (50 μM), API2 (100 μM), or LY294002 (50 μM) for 1 h (**E**). The quantification results of the band density were labeled above the bands.

Thus we next focused on the kinase function of PGK1, and constructed a PGK1 kinase-deficient mutant, T378P (Fig. 2A) [34]. We performed in vitro kinase assay using GST-PRAS40 and purified recombinant His-PGK1 or His-PGK1 T378P. The GST-PRAS40 incubated with His-PGK1 but not His-PGK1 T378P was phosphorylated, which was detected by anti-p-PRAS40 Thr246 antibody. This data suggest that PGK1 phosphorylates PRAS40 directly (Fig. 2B). We next found that compared with the empty vector-introduced cells, PRAS40 phosphorylation was markedly enhanced in wild-type PGK1-introduced cells. However, PRAS40 phosphorylation level in PGK1 T378P-introduced cells was lower than that in wild-type PGK1-introduced cells (Fig. 2C, D, S3C). Therefore Thr378 kinase site of PGK1 is important for PRAS40 phosphorylation.

Since most Thr246 phosphorylation of PRAS40 is considered as the result of AKT activation, and PGK1 overexpression also resulted in the upregulation of AKT phosphorylation, we wondered whether AKT mediates the PRAS40 phosphorylation by PGK1. We treated cells with rapamycin (mTOR inhibitor), LY294002 (PI3K inhibitor), or API2 (AKT inhibitor), and clarified that although PRAS40 phosphorylation levels were downregulated by LY294002 and API2, they were still higher in PGK1-overexpressing cells than empty vector-introduced cells, implying that PGK1 phosphorylates PRAS40 independently of AKT pathway (Fig. 2E). We previously reported that PRAS40 depletion led to the downregulation of AKT pathway [16], therefore the increase of AKT phosphorylation observed in PGK1-overexpressing cells might be caused by PRAS40 phosphorylation upregulation.

### PRAS40 mediates the promotion of cellular proliferation by PGK1

**Fig. 3:**
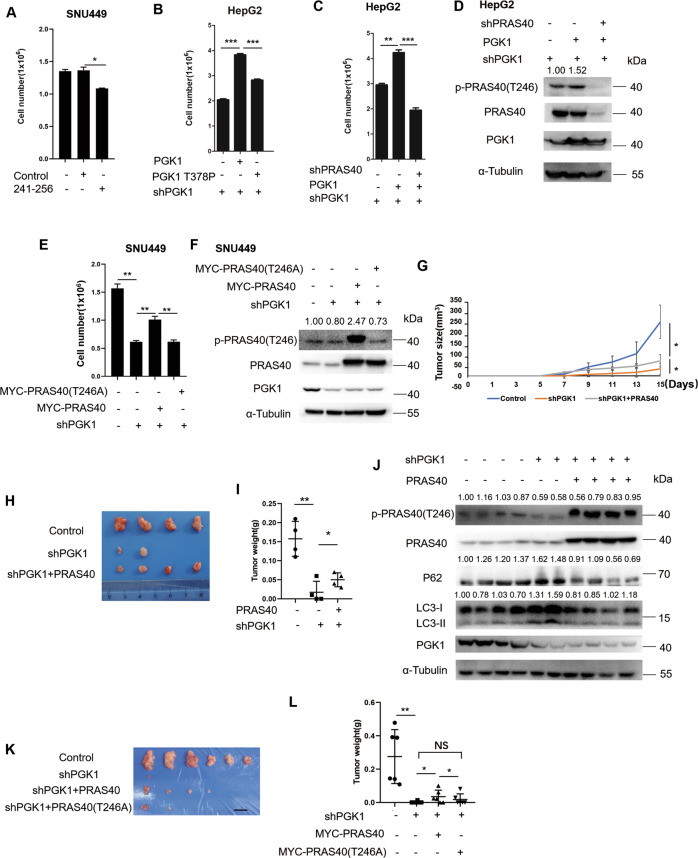
PGK1 induces cell proliferation via PRAS40 phosphorylation. **A, B** Cell counting for the indicated cells. The cells were treated with peptide control or peptide 241–256 (10 μM) (**A**). The cells depleted with PGK1 were introduced with empty vector, shRNA-resistant FLAG-PGK1, or FLAG-PGK1 T378P expression vector (**B**). **C, D** Cell counting and western blotting for the PGK1-depleted cells reintroduced with control, shRNA-resistant FLAG-PGK1 expression vector, or PRAS40 shRNA together with shRNA-resistant FLAG-PGK1 expression vector. **E, F** Cell counting and western blotting for the cells introduced with control, PGK1 shRNA (shPGK1), PGK1 shRNA together with Myc-PRAS40-expression vector, or PGK1 shRNA together with Myc-PRAS40 T246A expression vector. **G–J** HepG2 cells introduced with control, PGK1 shRNA, or PGK1 shRNA together with FLAG-PRAS40-expression vector, were injected into nude mice (*n* = 4). Tumor volumes were recorded every 2 days (**G**). On day 16, tumors were dissected and acquired. Images were taken (**H**), tumors were weighed (**I**), and the tumor tissue lysates were applied to western blotting (**J**). **K, L** HepG2 cells introduced with control, PGK1 shRNA, PGK1 shRNA together with Myc-PRAS40-expression vector, or PGK1 shRNA together with Myc-PRAS40 T246A expression vector were injected into nude mice (*n* = 6). On day 21, tumors were dissected and acquired. Images were taken (**K**), and tumors were weighed (**L**). Scale bar, 10 mm. Data represent the mean ± SD. *P < 0.05; **P < 0.01; ***P < 0.001. The quantification results of the band density were labeled above the bands.

Since peptide 241–256 treatment impaired cell growth and PRAS40 phosphorylation notably (*P* < 0.01–0.05, Fig. 1I, 3A, S2E, S3A, B), we next investigated the relationship of cell growth and the phosphorylation of PRAS40 induced by PGK1. Cellular proliferation was remarkably raised in wild-type PGK1-introduced cells, whereas PGK1 T378P-introduced cells showed a less upregulation of cell growth (*P* < 0.01, Fig. 3B, S3D).

Thus we hypothesize that the biological activities of PRAS40 phosphorylation by PGK1 are to mediate the PGK1-promoted cell growth. When PGK1 was reintroduced in PGK1-depleted cells, cell growth and PRAS40 phosphorylation level were increased significantly compared with the control cells, which was again reversed remarkably by PRAS40 knockdown (*P* < 0.01, Fig. 3C, D, S3E, F). After PGK1 was depleted, cell growth and PRAS40 phosphorylation were repressed notably compared with the control cells, while partly restored by PRAS40 overexpression. However, the introduction of PRAS40 T246A, a PRAS40 phosphorylation dead plasmid, did not show any alteration on the cell growth inhibition by PGK1 depletion (*P* < 0.01, Fig. 3E, F, S3G, H). Thus PRAS40 locates downstream of PGK1 signaling, and PGK1 contributes to cell growth via phosphorylating PRAS40 at Thr246 in vitro.

We further explored the possibility that PRAS40 overexpression reverses the cell growth repressed by PGK1 knockdown in mice (Fig. 3G-L). In contrast to that all the xenograft tumors formed in control group, only 2 (*n* = 4 in Fig. 3G-J) or 1 (*n* = 6 in Fig. 3K, L) tumor formed in PGK1-knockdown group (*P* < 0.01). When the PGK1-knockdown cells reintroduced with PRAS40-expression vector were injected, four tumors formed (*n* = 4 in Fig. 3G-J; *n* = 6 in Fig. 3K, L), and the tumor volume and weight were partly restored (*P* < 0.05). However, only two tumors formed in shPGK1+Myc-PRAS40 T246A group, and the tumor volume and weight were downregulated greatly compared to that of the shPGK1+Myc-PRAS40 group (*P* < 0.05), but not different from that of the PGK1-knockdown group (Fig. 3K, L). In addition, similar to the results in vitro, PRAS40 phosphorylation was downregulated remarkably in PGK1-knockdown tumor xenografts (Fig. 3J). These data suggest that PGK1 knockdown significantly suppressed PRAS40 phosphorylation, inhibiting tumor growth in vivo.

### PGK1 represses autophagy-mediated cell death

In the xenograft samples, LC3-II expression was upregulated when PGK1 was depleted, which was partially restored by PRAS40 overexpression (Fig. 3J). These results implied that autophagy could be influenced by PGK1 and PRAS40 in vivo, correlating to cell proliferation.

**Fig. 4:**
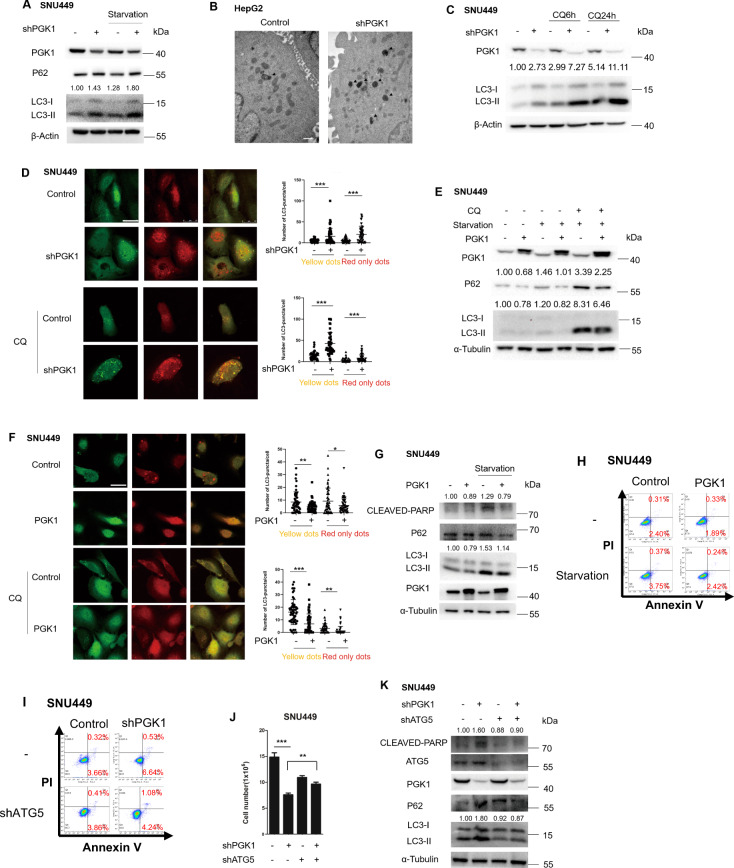
The association of PGK1 with autophagy-mediated cell death. **A–C** PGK1 shRNA was introduced into the indicated cells. Cells were cultured in normal or serum-free media (starvation) for 6 h, followed by western blotting analyses (**A**). Images were taken by transmission electron microscopy. Autophagosomes were signed with arrow heads. Scale bar, 500 nm (**B**). Cells were treated with or without CQ (10 μM) for indicated times, followed by western blotting analyses (**C**). **D** The indicated cells transfected with mRFP-EGFP-LC3 were treated with or without CQ (10 μM) for 6 h, and representative micrographs for LC3 puncta were shown. Scale bar, 25 μm. The puncta were counted in more than 50 cells of each treatment, and the numbers of autophagosomes (yellow dots) and autolysosomes (red only dots) per cell were plotted. **E–H** FLAG-PGK1 expression vector was introduced into the indicated cells. The indicated cells were cultured in normal or serum-free media (starvation) with or without CQ (10 μM) for 6 h (**E**). The indicated cells transfected with mRFP-EGFP-LC3 were treated with or without CQ (10 μM) for 6 h, and representative micrographs for LC3 puncta were shown. Scale bar, 25 μm. Quantification results were shown in right panel (**F**). The indicated cells were cultured in normal or serum-free media (starvation) for 24 h, followed by western blotting analyses (**G**). The indicated cells were stained with PI and Annexin V, followed by flow cytometry (**H**). **I–K** The cells transfected with control or ATG5 shRNA (shATG5) were introduced with or without PGK1 shRNA. Cells were stained with PI and Annexin V followed by flow cytometry (**I**), counted 48 h later (**J**) or cell lysate was applied to western blotting (**K**). Data represent mean ± SD. *P < 0.05; **P < 0.01; ***P < 0.001. The quantification results of the band density (LC3-II and CLEAVED-PARP) were labeled above the bands.

To clarify the effects of PGK1 on autophagy, we knocked down PGK1 and found a significant increase in LC3-II expression level, especially under starvation (Fig. 4A). The number of autophagosomes detected by transmission electron microscopy was also augmented in PGK1-knockdown cells, compared with control cells (Fig. 4B). To verify the autophagy process affected, the cells were treated with a lysosomal inhibitor CQ (chloroquine diphosphate). CQ-induced LC3-II accumulation (autophagy flux) was also increased by PGK1 knockdown (Fig. 4C). LC3 puncta formation in mRFP-EGFP-LC3 introduced cells was examined. The average number of the RFP^+^EGFP^+^ LC3 puncta (yellow dots) representing autophagosomes in each cell was much more in PGK1-knockdown cells (15.51) than control cells (5.02), while the average number of the RFP^+^EGFP^−^ LC3 puncta (red only dots) representing autolysosomes in each cell was also much more in PGK1-knockdown cells (20.27) than control cells (6.07). In addition, CQ treatment resulted in more autophagosomes (yellow dots) and autolysosomes (red only dots) in PGK1-knockdown cells (*P* < 0.001, Fig. 4D), indicating that PGK1 knockdown mainly promotes autophagosome formation instead of blocking LC3-II degradation. When PGK1 was overexpressed, LC3-II level was downregulated, which was enhanced by starvation and CQ treatment (Fig. 4E). PGK1 overexpression decreased the numbers of both autophagosomes (yellow dots) and autolysosomes (red only dots) no matter with or without CQ treatment (Fig. 4F). Therefore PGK1 inhibits autophagy.

Although p62 is the substrate of autophagy, it has been reported to be enrolled in apoptosis [35]. p62 was increased by PGK1 depletion while decreased by PGK1 overexpression, and cleaved poly ADP-ribose polymerase (PARP) were also downregulated in PGK1-overexpressed cells, which was enhanced by starvation (Fig. 4A, E, G). The alteration of p62 and cleaved PARP indicates the role of PGK1 in apoptosis. To verify the possibility further, we next examined apoptosis rate using flow cytometry. After 24 h starvation, the ratio of the early apoptotic cells, represented by that of the PI^−^/Annexin V^+^ cells, was increased to 3.75% in control cells, which were only 2.42% in PGK1-overexpressed cells (Fig. 4H). When PGK1 was depleted, the early apoptotic cells were increased to 6.64%, compared to 3.66% in control cells (Fig. 4I). To determine whether the apoptosis repressed by PGK1 was caused by autophagy, we depleted ATG5 (shATG5) to inhibit autophagosome formation. Accordingly the ratio of the early apoptotic cells was not altered significantly in PGK1-depleted cells (4.24%), compared to that in control cells (3.86%) (Fig. 4I). Similarly, the difference in the cellular proliferation rate between PGK1 knockdown and control cells was also significantly diminished by ATG5 knockdown (Fig. 4J). The levels of LC3-II, p62, and cleaved PARP upregulated in PGK1-knockdown cells declined significantly after ATG5 knockdown (Fig. 4K). These data suggest that PGK1 suppresses both autophagy and apoptosis, and the apoptosis inhibition by PGK1 is through downregulating autophagy. Thus PGK1 represses autophagy-mediated cell death.

### PGK1 represses autophagy-mediated cell death through PRAS40

**Fig. 5:**
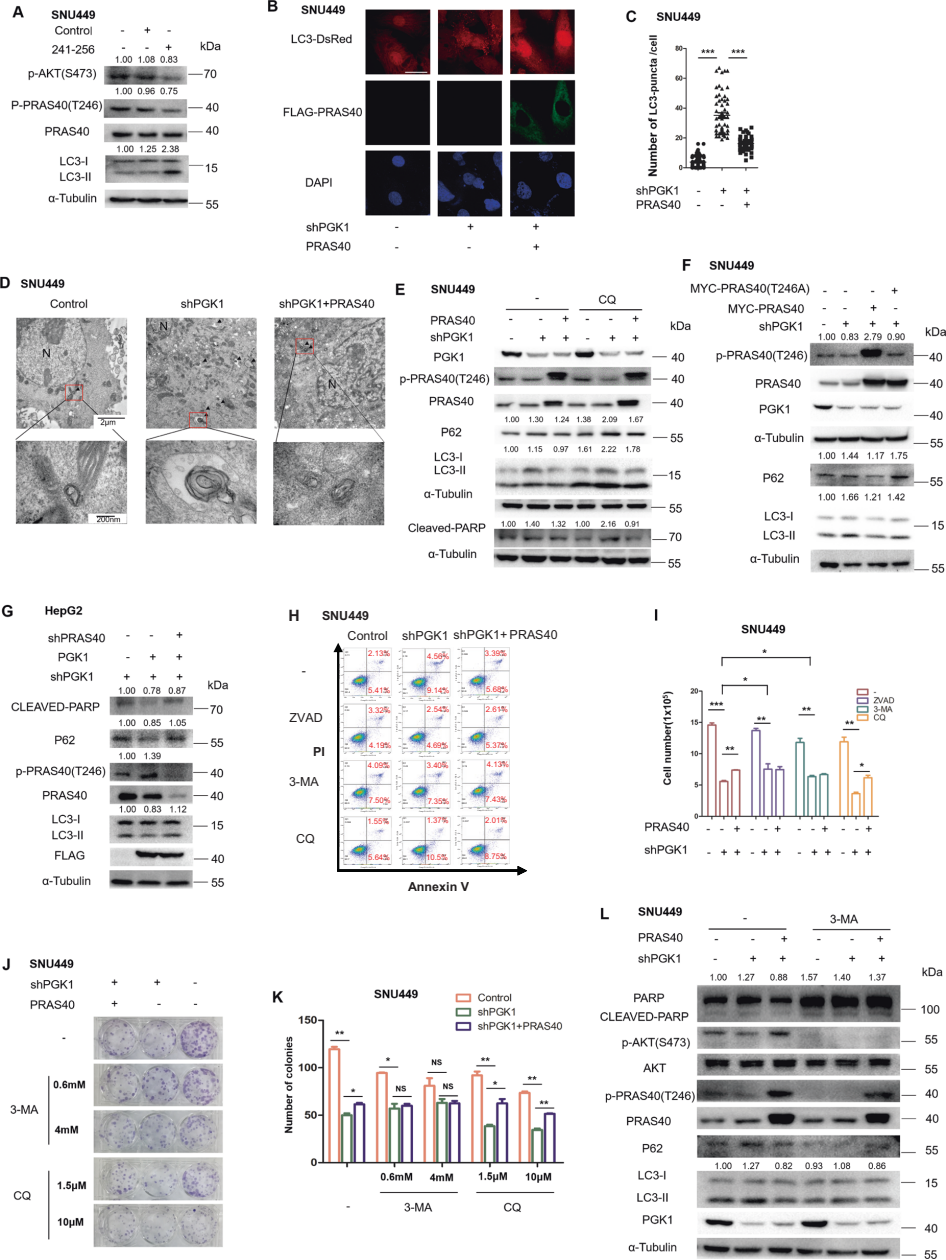
The involvement of PRAS40 in the repression of autophagy-mediated cell death by PGK1. **A** The indicated cells were treated with peptide control or peptide 241–256 (10 μM) for 6 h, followed by western blotting analyses. **B, C** Immunofluorescent staining was performed with anti-FLAG antibody in the indicated LC3-DsRed-expressing cells. Representative micrographs for LC3 (Red), FLAG-PRAS40 (Green), and nucleus (Blue) were shown. Scale bar, 25 μm (**B**). Quantification results for LC3 puncta from more than 50 cells of each treatment (**C**). **D** Images were taken by transmission electron microscopy. Autophagosomes were signed with arrow heads. N nucleus. Scale bar for upper panels, 2 μm; Scale bar for lower panels, 200 nm. **E–G** Western blotting analyses with the indicated antibodies. The indicated cells were treated with or without CQ (10 μM) for 24 h (**E**). **H–L** The indicated cells were treated with or without ZVAD (50 μM), 3-MA (2 mM), and CQ (10 μM). Cells were stained with PI and Annexin V, followed by flow cytometry 24 h later (**H**), counted 48 h later (**I**), applied to colony formation assay (**J, K**), or western blotting analyses (**L**). Data represent mean ± SD. *P < 0.05; **P < 0.01; ***P < 0.001. The quantification results of the band density (LC3-II and CLEAVED-PARP) were labeled above the bands.

To investigate the role of PRAS40 in the autophagy-mediated cell death by PGK1, we treated the cells with peptide 241–256 to disrupt the interaction of PGK1 and PRAS40. PRAS40 phosphorylation level declined accordingly, and LC3-II level rose notably (Fig. 5A, S4A). Next we overexpressed PRAS40 in PGK1-knockdown cells. The increase of the average number of LC3 puncta by PGK1 knockdown (35.18) compared with control cells (3.80) was reversed greatly by PRAS40 overexpression (16.20, *P* < 0.001, Fig. 5B, C). The number of the autophagosomes detected by transmission electron microscopy was also reversed to the level of control cells by PRAS40 overexpression (Fig. 5D). Simultaneously, the LC3-II levels upregulated by PGK1 depletion were restored after PRAS40 but not PRAS40 T246A introduction (Fig. 5E, F, S4B-D). On the contrary, the LC3-II levels downregulated in PGK1-overexpressed cells were increased significantly by PRAS40 knockdown (Fig. 5G). Thus PGK1 suppresses autophagosome formation through binding and phosphorylating PRAS40.

Furthermore, either PRAS40 overexpression or knockdown reversed the effects on the levels of p62 and cleaved PARP by PGK1 depletion or overexpression (Figs. 5E-G, S4B-D). Early apoptotic cells detected by flow cytometry were increased to 9.14% in PGK1 shRNA transfected cells (shPGK1) from 5.41% in the control cells, which was 5.68% in the cells PGK1 depleted while PRAS40 overexpressed (shPGK1 + PRAS40). The difference of early apoptosis among these cells vanished after the treatment with an apoptosis inhibitor ZVAD and an autophagy inhibitor 3-MA, while was enhanced by CQ treatment (Fig. 5H). In addition, the proliferation repression and the upregulation of p62 and cleaved PARP levels in PGK1-knockdown cells diminished significantly after the treatment with ZVAD or 3-MA, close to those in control cells or the cells depleted with PGK1 while overexpressed with PRAS40 (Figs. 5I-L, S4D). Thus PGK1 represses autophagy-mediated cell death through PRAS40.

### PGK1 shifts the binding partner from PRAS40 to Beclin1 under hypoxia

**Fig. 6:**
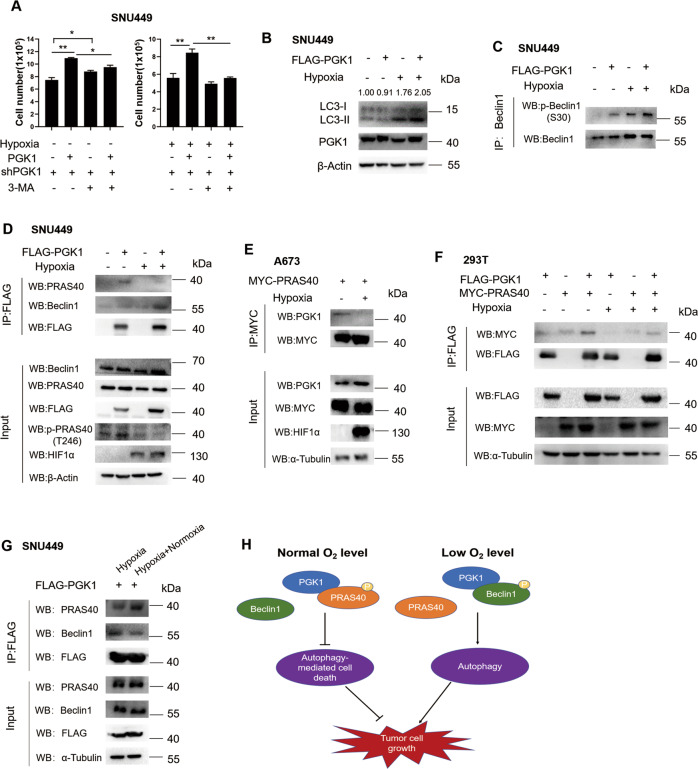
The relationship of PGK1 and PRAS40 under hypoxia. **A** Cell counting for the indicated cells. PGK1-depleted cells reintroduced with control or shRNA-resistant FLAG-PGK1 expression vector were treated with or without 3-MA and cultured under normal (21%, −) or lower oxygen level (1%, Hypoxia + ) for 48 h. Data represent mean ± SD. *P < 0.05; **P < 0.01. **B–F** The indicated cells were seeded and cultured under normal oxygen level for 24 h, and were kept under normal oxygen level (21%, −) or shifted to lower oxygen level (1%, Hypoxia + ) for another 24 h. Cell lysates were applied to western blotting analysis (**B**), or Co-IP followed by Western blotting with the indicated antibodies (**C–F**). **G** The PGK1-overexpressing cells were cultured under lower oxygen level for 24 h, and were kept under lower oxygen level (Hypoxia) or shifted to normal oxygen level (Hypoxia + Normoxia) for another 6 h. Cell lysates were applied to Co-IP followed by western blotting with the indicated antibodies. **H** Schematic graphs representing the working model of the interaction of PRAS40 and PGK1. The quantification results of the band density were labeled above the bands.

PGK1 has been reported to interact with and phosphorylate Beclin1 under hypoxia to induce autophagy, promoting the proliferation of glioblastoma cells [29]. Similarly, PGK1 overexpression induced the proliferation of SNU449 cells under hypoxia, which was reversed by 3-MA treatment (Fig. 6A). The western blotting analysis results showed that PGK1 overexpression exactly resulted in a significant increase of the levels of LC3-II under hypoxia. Further, PRAS40 phosphorylation induced by PGK1 overexpression under normoxia was significantly repressed by hypoxia treatment, whereas Beclin1 phosphorylation was remarkably increased by PGK1 overexpression especially under hypoxia (Fig. 6B, C, S5A). To investigate the relationship between PRAS40 or Beclin1 and PGK1 under different oxygen levels, we overexpressed PGK1 or PRAS40 and performed Co-IP (Fig. 6D-F, S5B). Surprisingly, when the cells were shifted to hypoxia from normoxia, PRAS40 in the complex with PGK1 was greatly less than that under normoxia, whereas Beclin1 in the complex with PGK1 was much more under hypoxia than that under normoxia as reported. Further, when the cells cultured under hypoxia were shifted to normoxia, PRAS40 co-precipitated with PGK1 was increased while Beclin1 co-precipitated with PGK1 was decreased significantly (Fig. 6G). Therefore PGK1 shifts the binding partner from PRAS40 to Beclin1 when the environmental oxygen level drops, switching the role from inhibiting autophagy-mediated apoptosis to improving autophagy.

## Discussion

In addition to that autophagy provides energy to promote cell growth, cell death related to autophagy also participates in both physiological and pathological events. A large body of evidence indicates that impaired autophagic activity suppresses tumor cell death, contributing to cell proliferation and tumorigenesis [36–40]. Thus autophagy functions as a double-edged sword in a context-mediated manner in tumorigenesis. Herein by screening the binding partners of PRAS40, we report that PRAS40 binds PGK1 and is phosphorylated by PGK1 (Fig. 1–3, S1–3). PGK1 promotes tumor cell proliferation by inhibiting autophagy-mediated cell death through PRAS40 under normoxia (Fig. 3–6, S3–5), while shifting the binding partner from PRAS40 to Beclin1 to improve autophagy under hypoxia (Fig. 6).

PRAS40 Thr246 phosphorylation has been detected in autophagy inhibition in monocytes [26]. Since PRAS40 is mainly phosphorylated by AKT at Thr246, the question that the involvement of PRAS40 phosphorylation in autophagy is only the result of AKT activation or represents other mechanisms still remains a miracle. Therefore we conducted the Co-IP followed by mass spectrometry to investigate the function network of PRAS40. PGK1 was clarified to bind PRAS40 at the region of amino acid 241–256 (Fig. 1, S1–2). Peptide 241–256 disrupted the interaction of PRAS40 and PGK1, and simultaneously repressed PRAS40 phosphorylation (Fig. 1I, 5A, S2E, S3A). PRAS40 was phosphorylated by wild-type PGK1 but not PGK1 T378P both in vivo and in vitro, suggesting that PGK1 phosphorylates PRAS40 as a kinase (Fig. 2, S3C). Further since in vitro kinase assay revealed that PGK1 directly phosphorylates PRAS40 (Fig. 2B), and AKT inhibitors did not impair the PRAS40 phosphorylation induced by PGK1, PGK1 should accordingly phosphorylate PRAS40 independently of AKT (Fig. 2E). We have shown that PRAS40 facilitates AKT phosphorylation through a positive feedback mechanism [15, 16], therefore AKT phosphorylation upregulated by PGK1 here should be mediated by PRAS40, which could explain the phenomena reported previously that PGK1 activates AKT pathway [33].

Peptide 241–256 which disrupted the interaction of PGK1 and PRAS40 hindered cellular proliferation (Fig. 3A, S3B); PGK1 T378P which could not phosphorylate PRAS40 lost the ability of cell growth promotion partly as well (Figs. 3B, S3D); PRAS40 knockdown completely reversed the growth promotion by PGK1 overexpression (Fig. 3C, S3E); while PRAS40 wild-type but not phosphorylation dead T246A could restore the cell growth suppressed by PGK1 depletion partly (Fig. 3E, S3G). Accordingly PRAS40 is a downstream factor of PGK1, and PGK1 phosphorylates PRAS40 to promote tumor cell proliferation. Since PRAS40 overexpression also partially restored the growth inhibition by PGK1 depletion, PRAS40 could possibly improve cell proliferation independently of PGK1 in part (Fig. 3E-I, S3G, H).

The next question is how PGK1 promotes cell growth through PRAS40 phosphorylation. PGK1 facilitates autophagy under glutamine deprivation or hypoxia [29], while we found that the number of autophagosomes, LC3-II, and autophagy flux was significantly upregulated by PGK1 knockdown under normoxia. Simultaneously, the levels of p62 and cleaved PARP were remarkably increased by PGK1 depletion. The opposite is also true (Fig. 4). Although p62 upregulation was reported in multiple cancers [41], it is also considered as crucial to cell death: p62 accumulated in *Atg5*^−/−^ tumor or virus-transformed cells induces DNA damage in response to glucose depletion or oxidative stress [42, 43]; Several anti-tumor drugs promote autophagy-dependent cell death through JNK-mediated p62 expression, showing the increase of both LC3-II and p62 [44–46]. Furthermore, apoptotic cells were notably decreased in PGK1-overexpressed cells while increased in PGK1-depleted cells, which could be reversed by autophagy inhibition. In addition, PGK1 knockdown could not increase the levels of p62 and cleaved PARP as well as LC3-II when autophagy was inhibited by either Atg5 knockdown or 3-MA treatment, therefore PGK1 suppresses autophagy-mediated cell death (Fig. 4–5). Moreover, either PRAS40 depletion or overexpression reversed the effects of PGK1 overexpression or depletion on autophagy-mediated cell death (Fig. 5, S4), suggesting the crucial role of PRAS40 in PGK1-repressed autophagy-mediated cell death, which could explain the mechanism for PGK1 promoting tumorigenesis.

PGK1 was previously reported to induce autophagy by phosphorylating Beclin1 under glutamine deprivation or hypoxia in cancer [29]. However, it is largely unknown whether PGK1 facilitates tumorigenesis under the environment of normoxia or normal nutrients. Different from that only the acetylated PGK1 binds Beclin1, wild-type PGK1 binds PRAS40 directly in vitro (Fig. 1E); PGK1 interacts with PRAS40 under normoxia, while associates to Beclin1 under hypoxia consistently with the previous report (Fig. 6). PGK1 suppresses autophagy-mediated cell death by phosphorylating PRAS40 under normoxia, while inducing autophagy through phosphorylating Beclin1 under hypoxia. Cancer cells exhibit different characteristics to adapt to the environment: Some cancer cells could survive under hypoxia or short of nutrients, there PGK1 works as an important rate-limiting glycolytic enzyme or phosphorylates Beclin1 to induce autophagy, supplying energy to cancer cells; while in cancer at an early stage or the cancer cells with plenty of oxygen or nutrient supply, PGK1 instead phosphosphorylates PRAS40 to inhibit autophagy-mediated cell death, promoting tumorigenesis (Fig. 6, S5). Thus PGK1 should work as a responser to oxygen alteration and switch to different partners depending on the oxygen level, contributing to tumorigenesis. Therefore suppressing PGK1/PRAS40 signaling could be possible to induce autophagy-mediated cell death under normoxia, which is anticipated to offer novel insights to target cancer.

## Materials and methods

### Cell lines and cell culture

Ewing’s sarcoma cell line A673, liver cancer cell lines HepG2 and SNU449 were purchased from ATCC, and were cultured for less than 6 months after resuscitation. HEK293T, tested for 8 STR loci and the amelogenin gene, and A673 were grown in Dulbecco’s Modified Eagle’s medium; HepG2 was grown in Eagle’s Minimum Essential Medium; SNU449 was grown in RPMI 1640, supplemented with 10% fetal bovine serum (FBS) and 2 mM glutamine. All the cell lines were incubated at 37°C with 5% CO_2_.

For starvation treatment, cells were cultured in serum-free media after the attachment; for hypoxia treatment, cells were cultured under 1% oxygen level after the attachment.

### Plasmids

pLKO.1-shPGK1- and pLKO.1-shPGK1-resistant expression vector pLEX-FLAG-PGK1 was kindly provided by Dr. Daming Gao [27]. pLEX-FLAG-PGK1 T378P was constructed by mutating Thr378 of PGK1 to Proline. pRK5-MYC-PRAS40 (Addgene plasmid #15476) and pLKO.1-PRAS40 (Addgene plasmid #15477) were gifts from Dr. Do-Hyung Kim [9], FLAG-PRAS40 was described previously [15]. Deletion mutants of PRAS40 were constructed by inserting the relevant domain of human AKT1S1 cDNA with a FLAG sequence at the N-terminus into EcoRI-BamHI site of pSin-EF2 vector. pLVX-LC3-DsRed was kindly provided by Dr. Quentin Liu [47].

### Antibodies

Antibodies were purchased for detection of p-PRAS40 (T246), PRAS40, p-AKT (S473), LC3, PARP, cleaved caspase 3, HIF1α, Beclin1 (Cell Signaling); PGK1, AKT, α-Tubulin, β-Actin, ATG5 (proteintech); MYC (MBL); p62 (Abcam); and p-Beclin1 (S30)(SAB).

### Co-IP

Cell extracts were incubated with anti-FLAG M2 Affinity Gel (Sigma), or anti-MYC affinity gel (MBL), or anti-PRAS40 antibody or control IgG together with protein A agarose (GE) at 4 °C overnight, and immunoprecipitates were then subjected to Western blotting assay.

### Mass spectrometry analysis

The immunoprecipitates were loaded on the SDS-PAGE, and stained with Coomassie blue. The bands were cut out, freeze-dried, reduced with DTT, and then acetone-precipitated. The samples were next trypsinized for 20 h at 37 °C, and peptides were extracted, finally analyzed by LC-MS/MS on a LTQ-Velos mass spectrometer (Thermo Fisher Scientific).

Proteins were identified by comparing the fragment spectra against those in the UniProt database using BioworksBrowser 3.3.

### GST pull-down assay

The lysates of the bacteria carrying GST or GST-PRAS40 were incubated at 4 °C for 1 h with glutathione-Sepharose beads (GE). The lysates of the bacteria carrying His-PGK1 were mixed with the beads immobilized with GST fusion proteins at 4 °C for another 1 h, and the precipitates were then subjected to western blotting assay.

### Immunofluorescence cytochemistry

Cells seeded on coverslips were fixed in 4% paraformaldehyde at room temperature for 30 min and permeabilized in 0.5% Triton X-100 in PBS for 10 min. Cells were then incubated with the indicated antibodies at room temperature for 1 h, followed by incubation with Alexa 488 or Alexa 594 labled secondary antibodies for 45 min at room temperature (Thermo fisher). Nuclei were stained by DAPI (1 μg/μl), and cells were visualized using a confocal microscope (Leica).

### Lentivirus production and transduction

Virus particles were harvested 48 h after expression vector transfection with packaging plasmid psPAX2 (a gift from Dr. Didier Trono, Addgene plasmid #12260) and envelope plasmid pMD2.G (a gift from Dr. Didier Trono, Addgene plasmid #12259) into HEK293T cells using Lipofectamine 2000 reagent (Thermo fisher). Cells were infected with lentivirus with the presence of 8 μg/ml Polybrene (Sigma).

### In vitro kinase assay

The lysates of the bacteria carrying empty vector, His-PGK1 or His-PGK1 T378P were incubated with Ni-NTA Sepharose beads (GE) at 4 °C for 1 h. After dialysis, the eluted His-tagged proteins were incubated with glutathione-Sepharose-bound GST-PRAS40 in kinase buffer (50 mM Tris-HCl [pH 7.5], 100 mM KCl, 50 mM MgCl2, 1 mM Na_3_VO_4_, 1 mM DTT, 5% glycerol, 0.5mMATP) at 30 °C for 30 min. The reactions were terminated by adding SDS-PAGE loading buffer, and the mixtures were then subjected to western blotting assay.

### Cell growth assay

2 × 10^4^–1 × 10^5^ cells were seeded into six-well plates in triplicate. Cell proliferation was determined by cell counting 48–72 h later using Countess II (Thermo fisher).

### Peptide synthesis

Peptides were synthesized by linking FITC and a cell-penetrating peptide [48] to the target sequence (CHINESE PEPTIDE). Peptide 240–256: FITC-CYGRKKRRQRRRC-RPRLNTSDFQKLKRKY; peptide control: FITC-CYGRKKRRQRRRC-YRKPRRKNLTKNQSFD.

### In vivo tumorigenicity assay

All animal maintenance and procedures were carried out in strict accordance with the recommendations established by the Animal Care and Ethics Committee of Dalian Medical University. The protocol was approved by the Animal Care and Ethics Committee of Dalian Medical University. In the animal study, all efforts were made to minimize the suffering of mice.

All animals were maintained and animal experiments were conducted in the specific-pathogen-free (SPF) Laboratory Animal Center of Dalian Medical University. The mice were divided randomly into different groups. HepG2 cells (1.5–2 × 10^6^) introduced by negative control, PGK1 shRNA, PGK1 shRNA together with PRAS40-expression vector, or PGK1 shRNA together with PRAS40 T246A expression vector containing virus were injected subcutaneously into the two posterior flanks of male BALB/c nude mice (*n* = 4 or 6) (Vital River, Beijing). When tumors of the mice injected with negative control-introduced cells were detectable by palpation, we started to record the sizes of tumors measured by a caliper. The tumor volume was calculated by the formula V = 1/2 (width^2^ × length). On day 16 or 21 after tumor cell injection, all mice were sacrificed, and tumors were dissected by blinded technicians and weighed.

### Transmission electron microscopy (TEM)

Cell pellets were fixed in 2.5% glutaraldehyde followed by 1% osmium tetroxide (OsO4). After being embedded in reductive resin, sections (80 nm) were obtained, and stained with uranyl acetate and lead nitrate. The sections were observed using the transmission electron microscope (JEM-2000EX).

### Monitoring autophagy flux

Cells were transfected with mRFP-EGFP-LC3, and were treated with or without 10μM CQ for 6 h. Then Images were acquired using a confocal microscope (Leica TCS SP5 ×). RFP^+^EGFP^+^ LC3 puncta (yellow dots) were considered as autophagosomes, while RFP^+^EGFP^−^ LC3 puncta (red only dots) were considered as autolysosomes [1, 49].

### Statistical analyses

Data are presented as mean ± standard deviation (SD). Differences between groups were assessed by two-sided Student’s *t*-test. All experiments were repeated thrice. *P* < 0.05 was considered statistically significant. SPSS 18.0 software was used for all statistical analyses.

## Supplementary information

supplement data

Related Manuscript File

## Data Availability

All of the data in this study are available from Lin Huang on reasonable request.

## Materials availability

All of the materials in this study are available from Lin Huang on reasonable request.
