# Supplementary material for: PGK1 represses autophagy-mediated cell death to promote the proliferation of liver cancer cells by phosphorylating PRAS40
Source: Cell Death Dis. 2022 Jan 20;13(1):68. doi: 10.1038/s41419-022-04499-0 (PMC8776853; doi:10.1038/s41419-022-04499-0)
Supplement: Supplementary file 1 — supplement data [file 41419_2022_4499_MOESM1_ESM.pdf]

Extended Data

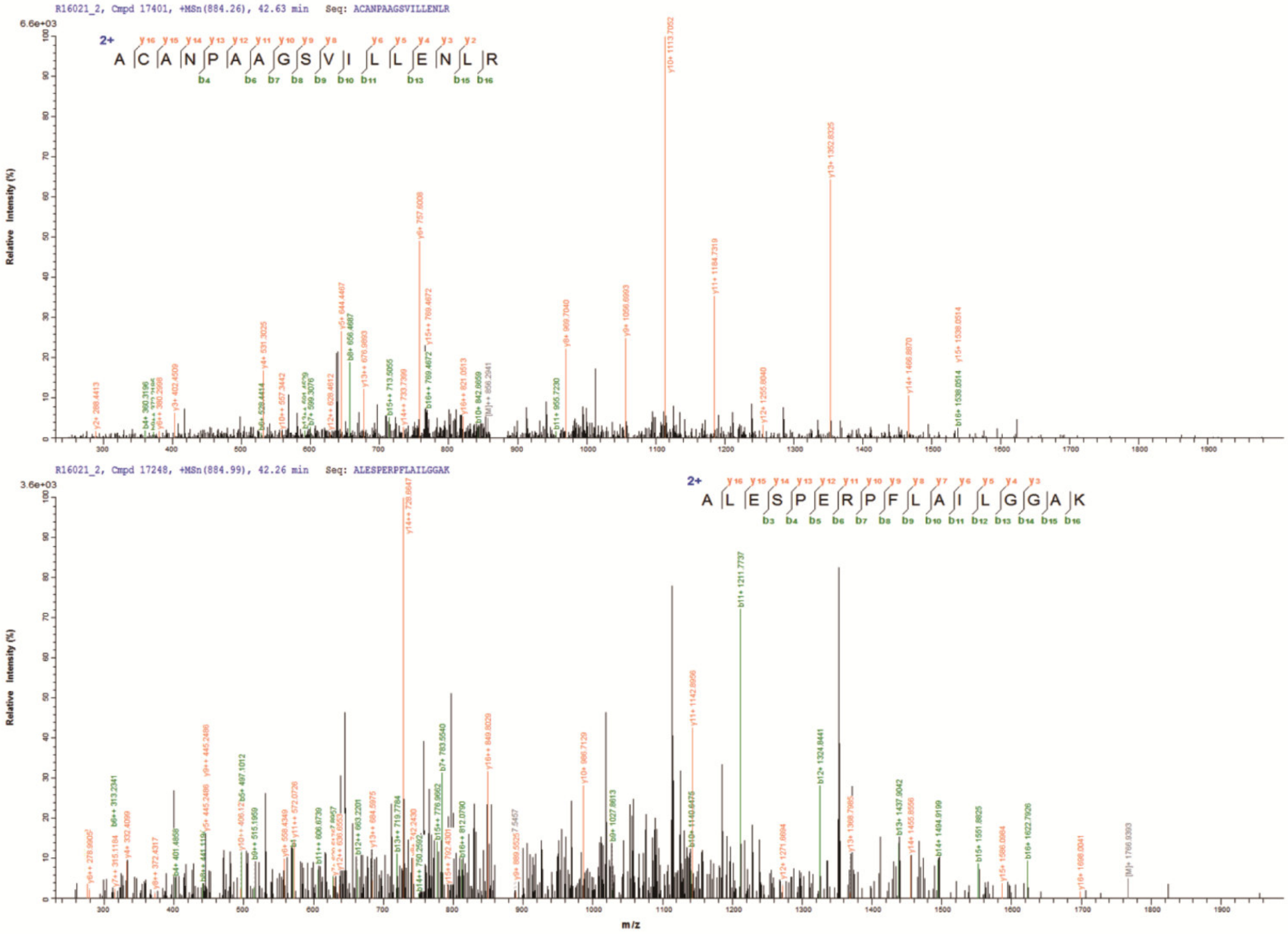

R16021_2, Cmpd 20902, +MSn(1053.68), 51.19 min Seq: QIVWNGPVGVFEWEAFAR

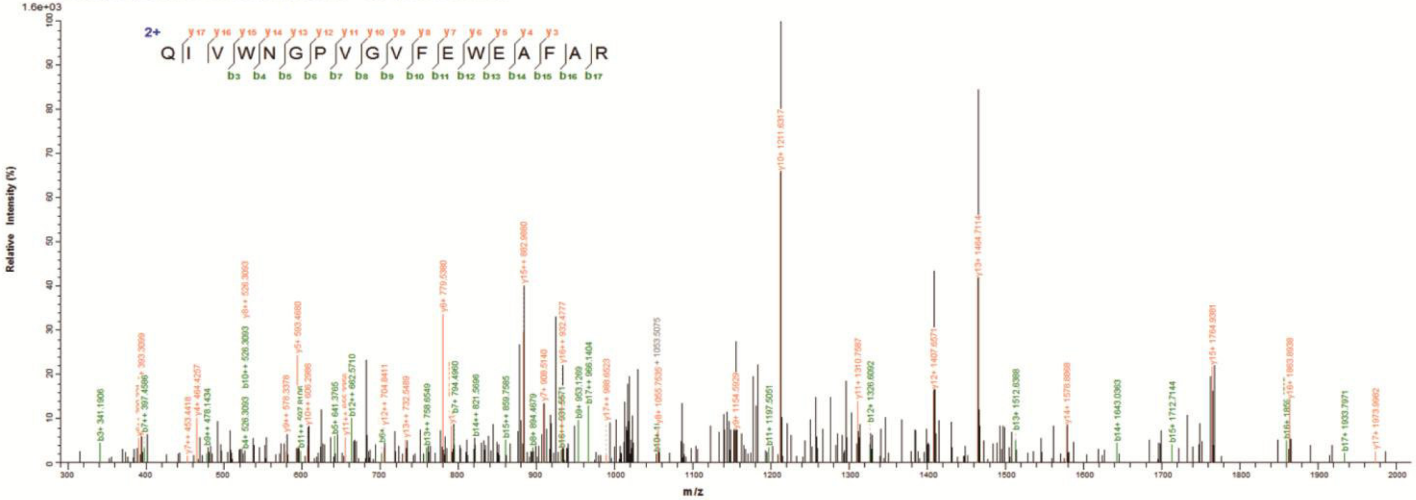

R16021_2, Cmpd 16035, +MSn(984.72), 39.28 min Seq: VLNNMEIGTSLFDEEGAK

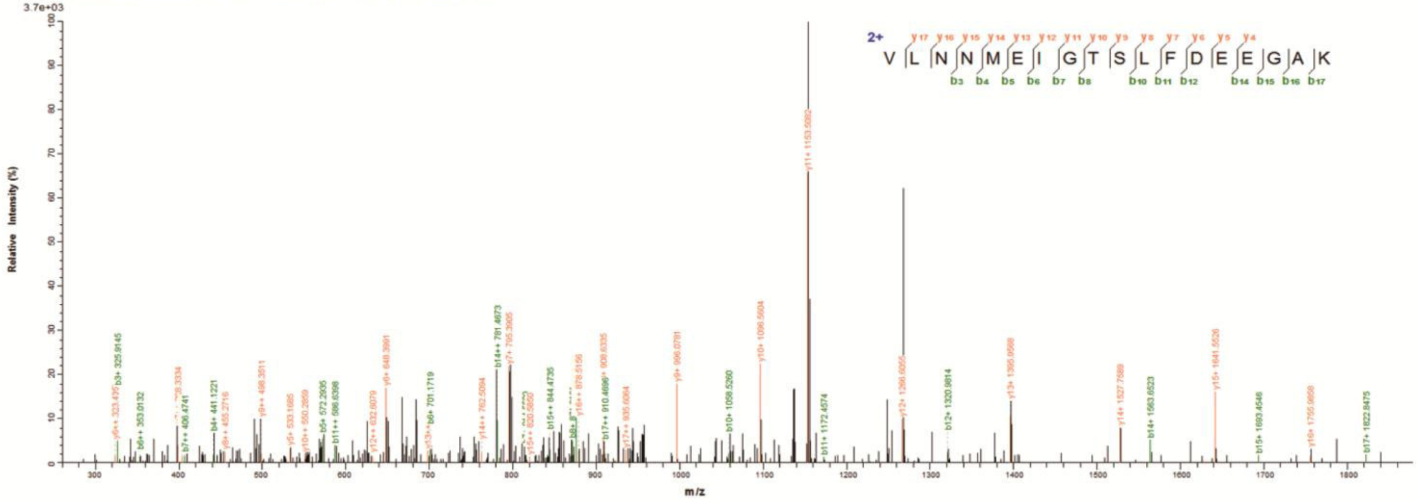

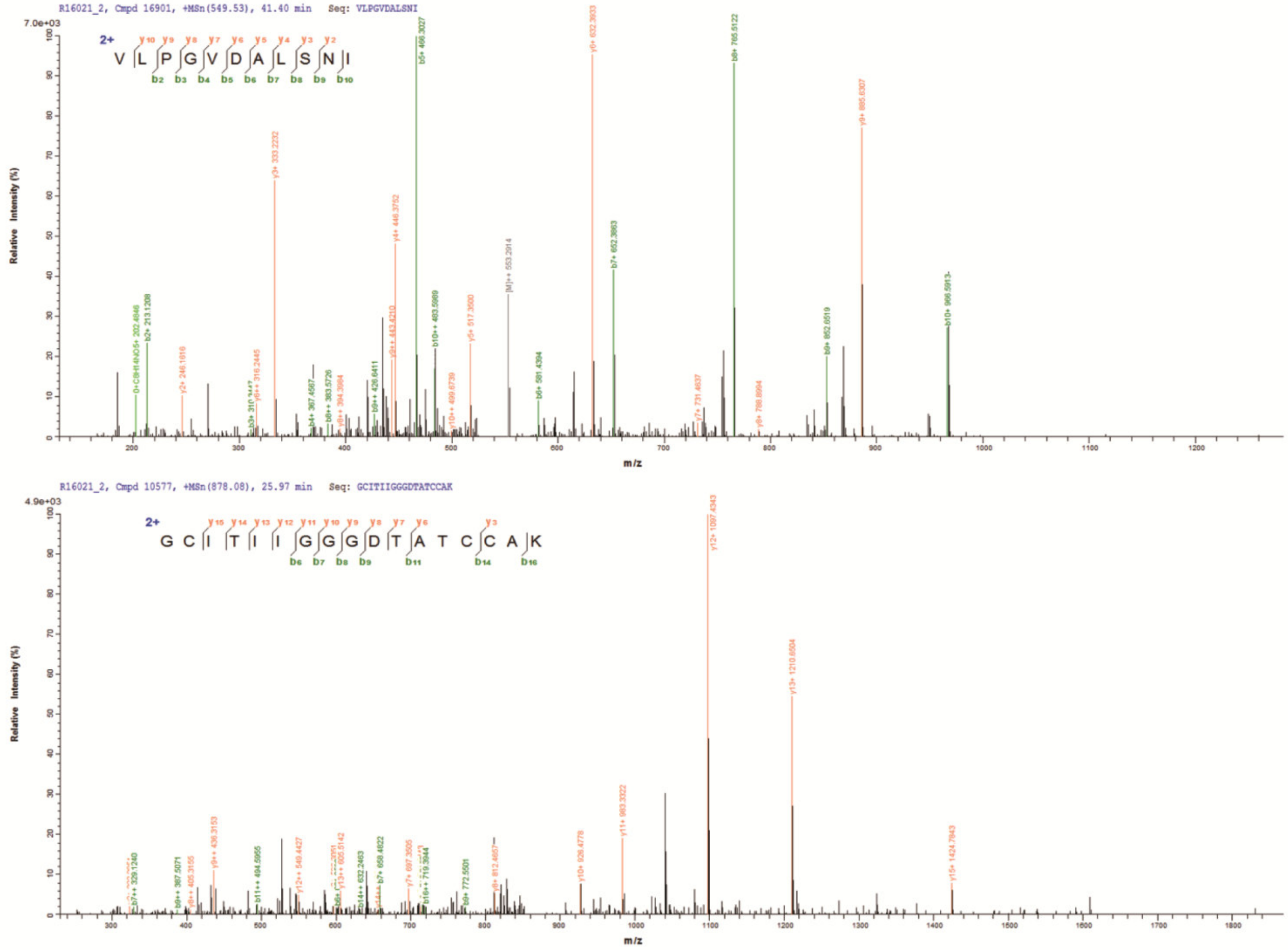

Figure S1. MS/MS figures for each unique peptide of PGK1 hit by mass spectrometry.

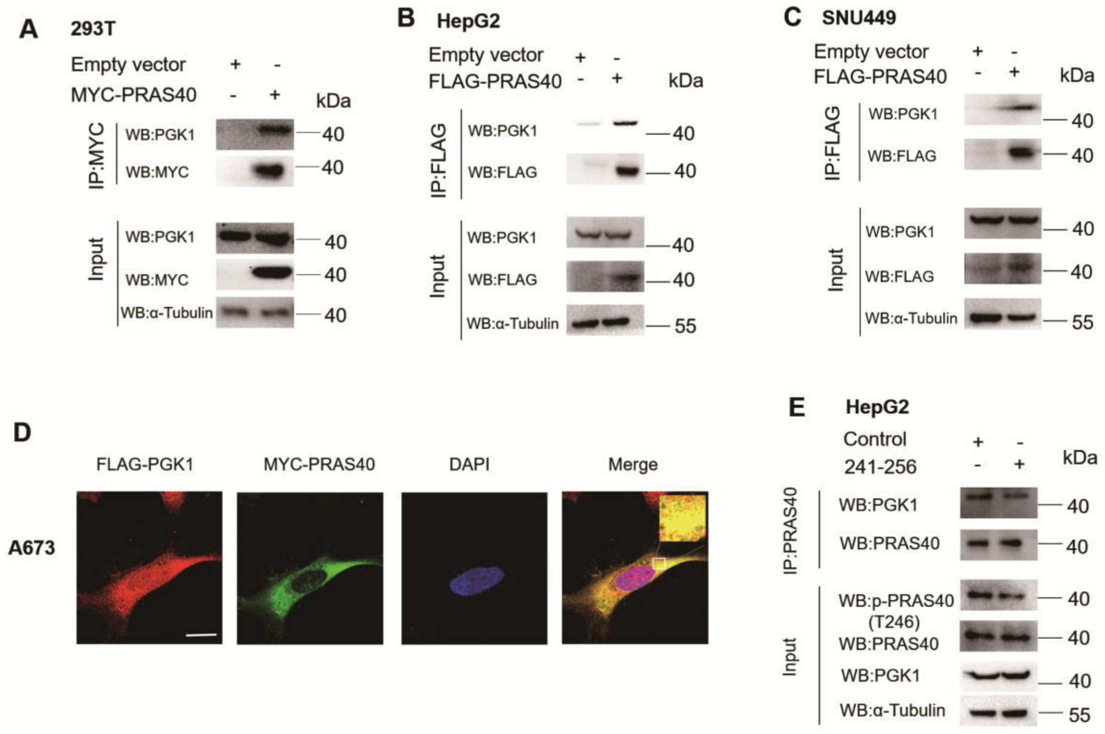

Figure S2. The binding of PRAS40 and PGK1. (A-C) Co-IP with the indicated antibodies in the indicated cells introduced with PRAS40 expression vector or empty vector. (D) Immunofluorescent staining with anti-FLAG and anti-Myc antibodies in the cells introduced with FLAG-PGK1 and Myc-PRAS40. Scale bar, 10μm. (E) Cells were treated with peptide control or peptide 241-256 (10μM). Six hours later, cell lysates were applied to the Co-IP with anti-PRAS40 antibody followed by Western Blotting analyses with the indicated antibodies.

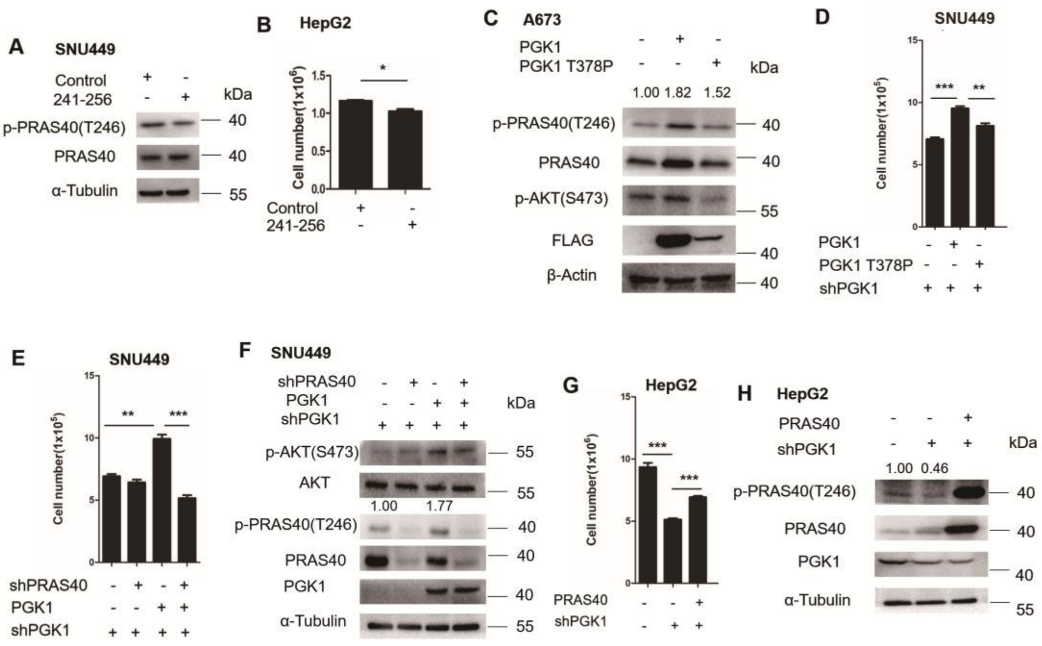

Figure S3. PGK1 induces cell proliferation via PRAS40 phosphorylation. Western blotting and cell counting for the indicated cells. (A-B) Cells were treated with peptide control or peptide 241-256 (10μM). Six hours later, cell lysates were applied to Western Blotting analyses with the indicated antibodies (A). Forty eight hours later, cells were counted (B). (C-D) A673 cells (C) or SNU449 cells depleted with PGK1 (D) were introduced with empty vector, shRNA-resistant PGK1 or PGK1 T378P expression vector. (E-F) PGK1-depleted cells were reintroduced with control, PRAS40 shRNA (shPRAS40), PGK1 expression vector (PGK1) or PRAS40 shRNA together with PGK1 expression vector. (G-H) Cells were introduced with control, PGK1 shRNA (shPGK1), or PGK1 shRNA together with PRAS40 expression vector. Data represent mean ± SD of three independent experiments. *, *P*<0.05; **, *P*<0.01; *** *P*<0.001.

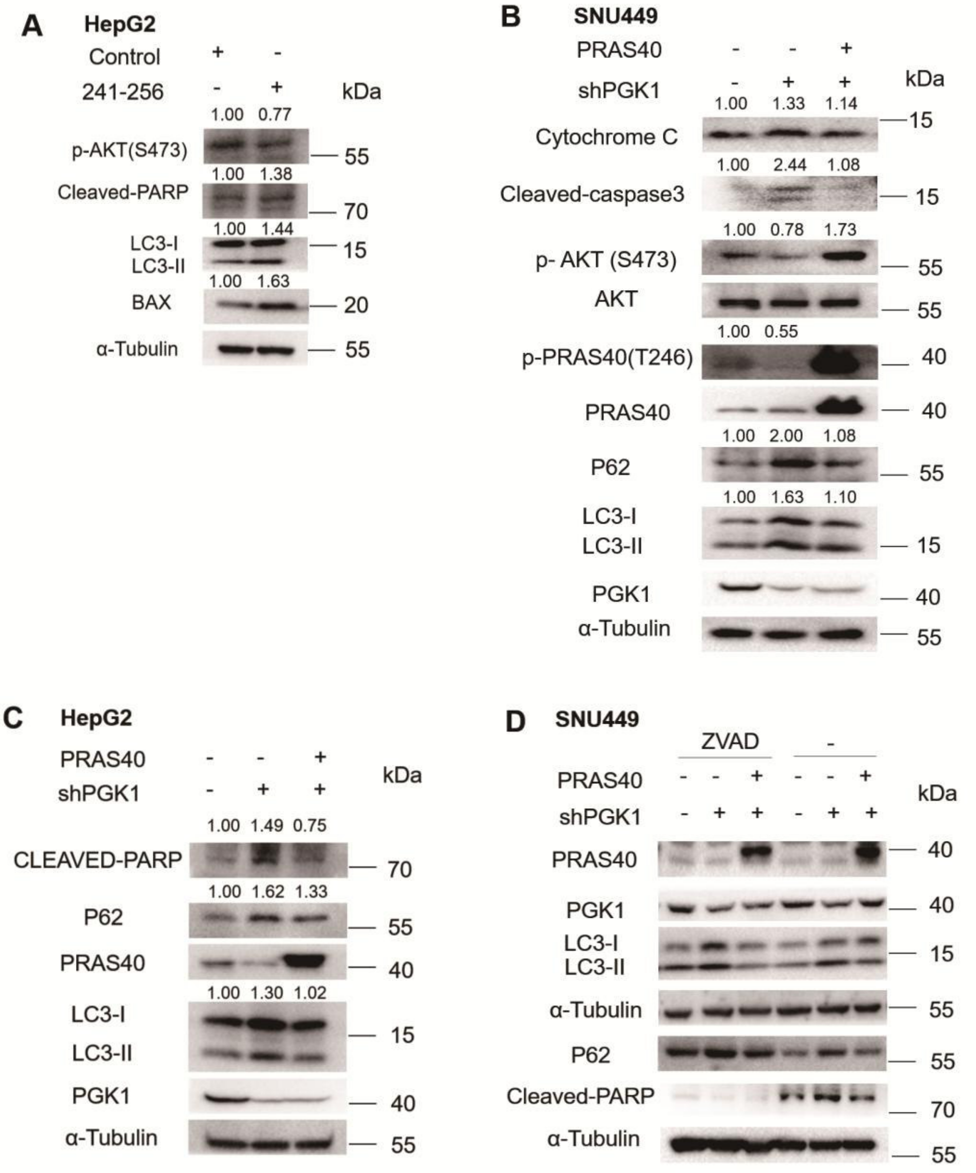

Figure S4. Autophagy-dependent cell death repressed by PGK1 through PRAS40. Western blotting for the indicated cells. (A) The cells were treated with peptide control or peptide 241-256 (10µM) for 6 hrs. (B-D) The cells were introduced with control, PGK1 shRNA (shPGK1) or PGK1 shRNA together with PRAS40 expression vector. Cells were treated with or without ZVAD (50µM).

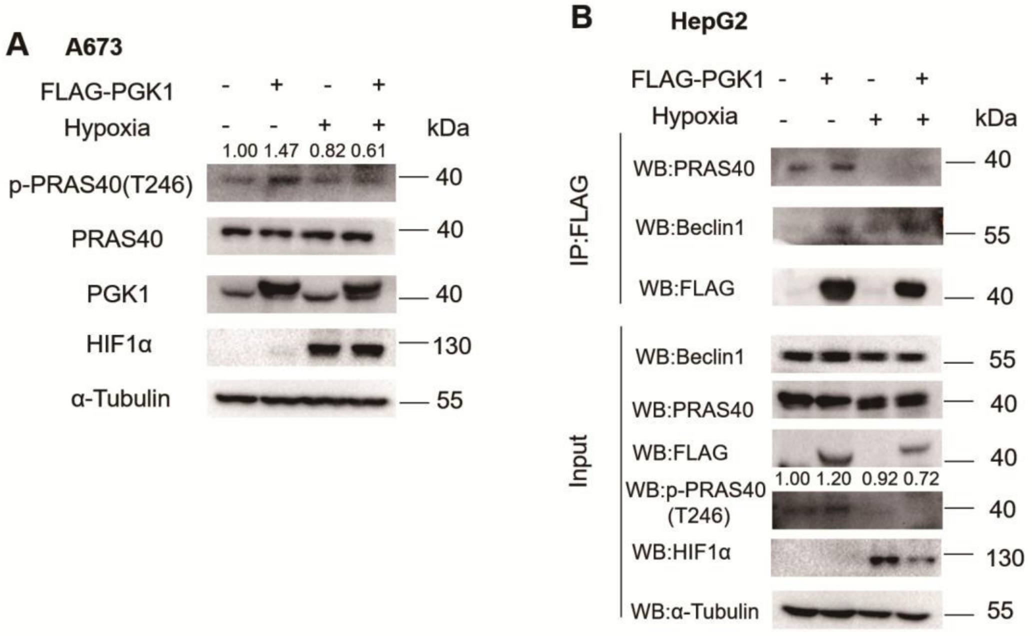

Figure S5. The relationship of PGK1 and PRAS40 under hypoxia. Western blotting and Co-IP in the cells introduced with empty vector or FLAG-PGK1 under normal oxygen level (21%, -) or lower oxygen level (1%, +).
